# Conventional early infant diagnosis in Lesotho from specimen collection to results usage to manage patients: Where are the bottlenecks?

**DOI:** 10.1371/journal.pone.0184769

**Published:** 2017-10-10

**Authors:** Appolinaire Tiam, Michelle M. Gill, Heather J. Hoffman, Anthony Isavwa, Mafusi Mokone, Matokelo Foso, Jeffrey T. Safrit, Lynne M. Mofenson, Thorkild Tylleskär, Laura Guay

**Affiliations:** 1 Centre for International Health, University of Bergen, Bergen, Norway; 2 Medical and Scientific Affairs, Elizabeth Glaser Pediatric AIDS Foundation, Maseru, Lesotho; 3 Research, Elizabeth Glaser Pediatric AIDS Foundation, Washington DC, United States of America; 4 Milken Institute School of Public Health, George Washington University, Washington DC, United States of America; 5 Research Alliances, International AIDS Vaccine Initiative, New York, New York, United States of America; Waseda University, JAPAN

## Abstract

**Introduction:**

Early infant diagnosis is an important step in identifying children infected with HIV during the perinatal period or in utero. Multiple factors contribute to delayed antiretroviral treatment initiation for HIV-infected children, including delays in the early infant HIV diagnosis cascade.

**Methods:**

We conducted a retrospective study to evaluate early infant diagnosis turnaround times in Lesotho. Trained staff reviewed records of HIV-exposed infants (aged-6-8 weeks) who received an HIV test during 2011. Study sites were drawn from Highlands, Foothills and Lowlands regions of Lesotho. Central laboratory database data were linked to facility and laboratory register information. Turnaround time geometric means (with 95% CI) were calculated and compared by region using linear mixed models.

**Results:**

1,187 individual infant records from 25 facilities were reviewed. Overall, early infant diagnosis turnaround time was 61.7 days (95%CI: 55.3–68.7). Mean time from specimen collection to district laboratory was 14 days (95%CI: 12.1–16.1); from district to central laboratory, 2 days (95%CI 0.8–5.2); results from central laboratory to district hospital, 23.3 days (95%CI: 18.7–29.0); from district hospital to health facility, 3.2 days (95%CI 1.9–5.5); and from health facility to caregiver, 10.4 days (95%CI, 7.9–13.5). Mean times from specimen transfer to the central laboratory and for result transfer from central laboratory to district hospital were significantly shorter in the Lowlands Region (0.9 and 16.2 days, respectively), compared to Highlands Region (6.0 [P = 0.030] and 34.3 days [P = 0.0099]. Turnaround time from blood draw to receipt of results was significantly shorter for HIV infected infants compared to HIV uninfected infants [p = 0.0036] at an average of 47.1 days (95%CI: 38.9–56.9) and 62 days (95%CI: 55.9–68.7) respectively. Of 47 HIV-infected infants, 36 were initiated on antiretroviral therapy at an average of 1.3 days (95%CI: 0.3, 5.7) after caregiver received the result.

**Conclusion:**

HIV-infected infants received results earlier and were rapidly initiated on antiretroviral therapy once the result was delivered to caregiver. However, average early infant diagnosis turnaround time was two months; the longest period of delay was transfer of results from central laboratory to district hospital. Turnaround time of results based on geographical regions or between hospitals and health centres varied but did not reach statistical significance.

## Introduction

Multiple factors contribute to delayed antiretroviral treatment (ART) initiation for HIV-infected children, including service delivery gaps in early infant HIV diagnosis (EID) [1–3]. Despite advances made in the field of prevention of mother-to-child transmission (PMTCT) of HIV, the average age of initiation of treatment for HIV-infected children is approximately five years [3,4]. Though there are multiple reasons for this, poor access to infant HIV testing and diagnosis is one key barrier. While EID access has improved, only 51% of the 1.6 million HIV-exposed African children had access to EID testing in 2015[5]. Approximately 23% of Lesotho’s population are living with HIV, making it among the highest per capita HIV infection rates in the world [4,5]. Of the approximately 310,000 individuals living with HIV in Lesotho, 13,000 are children < 15 years of age [6,7].

The Lesotho Ministry of Health (MOH) guidelines for EID are consistent with the World Health Organization (WHO) guidelines, recommending that the first virological test for infants exposed to HIV should be conducted at or around 6 weeks following birth, and all infants diagnosed with HIV should be started on ART immediately irrespective of CD4 count [8,9]. However, there are still gaps in coverage for EID/Early Infant Treatment (EIT) services in Lesotho, especially in rural areas [10]. In a study in Uganda, it was found that EID coverage was 16% (101/636); 4.5% (8/179) and 20.3% (93/457) in rural and urban health facilities respectively [11]. Similar gaps were reported in South Africa [12].

As of 2013, more than 50% of exposed/infected children did not receive their results within 2–4 weeks and approximately 30% of infants were lost to follow-up (either not enrolled into care or retained in care) in Lesotho [9]. The pathway from sample collection to results received was multifaceted and a delay in one point influenced the overall efficiency and TAT of the process. At the time of the study, the utilized pathway had several steps that could cause significant delays. While EID can be conducted using a variety of virologic assays, in Lesotho, EID is conducted using a DNA polymerase chain reaction (DNA-PCR) assay. At the six-week postpartum visit, HIV-exposed infants received a physical examination and immunizations and a dried blood spot (DBS) sample was taken for DNA-PCR testing to determine the child’s HIV status. Collected DBS samples were transported from health facilities to the district laboratory (referred to as the district hub); for visits at district hospitals, the laboratory was on the same campus. The specimen was then sent to laboratory headquarters (central laboratory) for personnel to decide whether the specimen should be processed at the central laboratory in Maseru or sent to another laboratory in South Africa for processing. This decision was based on the origin and quality of the sample. The national laboratory in South Africa operated an automated system while at the time of the study, the Lesotho National reference laboratory operated a manual system that was replaced by an automated system. For specimens to be sent to South Africa, the blood spot must be within the demarcated circle otherwise it had to be processed in Maseru. In addition, high volume facilities such as hospitals had their specimens sent to South Africa. At the time of the study, the proportion of specimens sent to South Africa for processing varied from one third to about half. Once processed, test results were delivered back to the district hub and then returned to the health facility. Caregivers were typically advised to return to the health facility after four weeks from the date of child’s blood draw. However, when some caregivers returned and test results were not yet available, they might not return to the health facility again to collect their infants’ test results. Additionally, some patients did not return to the health facility for other reasons and were then lost to follow-up. Furthermore, for children who were diagnosed as HIV-infected, test results were sent electronically to health facilities through 3G mobile internet and short message service (SMS). Community health workers actively track HIV-infected children back to the health facility within seven to ten days to enable them to be initiated on ART in accordance to national guidelines.

As the guidelines call for testing and treating all those living with HIV including children, there is an increasing need to scale up HIV diagnosis especially among infants and children [9]. Lesotho has made great strides in rolling out a national PMTCT program and the current mother to child transmission of HIV (MTCT) rate is estimated at 5.9% [7]. There are limited data that analyze the EID cascade to demonstrate barriers to efficient EID in sub-Saharan Africa, especially in a country with diversified topography like Lesotho. This paper describes the EID process and identifies bottlenecks within the EID pathway by tracking the length of time for each step in the cascade.

## Materials and methods

### Study design

We conducted a retrospective cohort study with the aim to describe the EID process in order to identify the barriers and delays within the EID pathway by tracking the length of time for each step. To determine where the delays existed between steps in the EID pathway, we estimated the average time intervals between the following time points:

- The 6-8-week HIV specimen is collected at the facility,
- The specimen is sent to the laboratory,
- The laboratory received the specimen,
- The laboratory processes the specimen and obtains results,
- The laboratory sends the results back to the facility,
- The facility receives the results from the laboratory,
- The facility contacts the caregiver,
- The caregiver receives the results,
- The HIV-infected infants are initiated on ART at study sites.

The variability for each time interval was estimated and compared between caregivers within sites (mean of the variances) and between sites (variance of the means). The characteristics of facilities were purposively predetermined based on topographic location as highlands, foothills and lowlands, which determine health seeking behaviour in Lesotho.[6] Unique characteristics of sites with the shorter time intervals were identified and compared to the sites with longer time intervals.

### Study population

Trained study staff abstracted data from the national laboratory database on all HIV-exposed infants who had a DNA-PCR EID test at 6–8 weeks of age from January to December 2011 in 25 sites from all 10 districts in Lesotho. Data abstracted from the database included infant age, test result, district hub and dates when the tracked specimen was received and processed. Using the child’s name and other key information, infants’ database records were linked to facility records from which their mothers received care. The records included the DNA-PCR EID test result, antenatal care (ANC), and ART registers and laboratory documentation. From these records, limited mother and infant demographics, mother and infant ARV regimens, and additional dates that documented the specimen and result along the EID pathway were abstracted. The documented dates reflected the physical receipt of the specimen and the results. During the study time, all sites were using 3G mobile internet and short message service (SMS) to communicate some of the results, especially results that were HIV-positive. The 3G mobile internet and the SMS systems were rolled out as part of project supported PEPFAR through the Elizabeth Glaser Pediatric AIDS Foundation (EGPAF). Once the results were sent to health facilities, electronic records as email were kept in the EGPAF head office in Maseru. Study staff also completed a data collection form which documented information about each facility, including characteristics, such as distance to district hub and mode of transport for samples, and strategies for identification and follow-up of mother-exposed infant pairs.

### Study sites

25 sites were included in this study. Of these 25 study sites, 11 were health centers that were purposively selected, and were defined as hard-to-reach.[6] Of these 11 hard-to-reach, 8 facilities were located in the highlands and presented a challenge during winter when roads are blocked with heavy snow fall. These 11 facilities were located in six districts, each representing the three geo-topographical areas of Lesotho as follows: Highlands (Mokhotlong, Qacha's Nek, Thaba Tseka); Foothills (Butha Buthe and Quthing); and Lowlands (Maseru). Program data showed that the hard-to-reach sites had longer TATs estimated at 6–8 weeks. An additional 14 sites were selected from all 10 districts of the country in order to represent more typical TATs. The topographical distribution of these 14 sites was as follows: Highlands (Mokhotlong, Qacha’s Nek, Thaba Tseka) 3 sites; Foothills (Butha Buthe, Leribe, Mohale’s hoek and Quthing) 6 sites; and Lowlands (Berea, Mafeteng, Maseru) 5 sites. The majority of these latter sites had estimated TATs of 2–4 weeks. Study sites included 16 health centers and nine hospitals.

### Data collection and analysis

Data were collected in March–April 2012 and entered into MS Access (2007–2010). The ANC identification number was used to link laboratory and facility records. Once records had been matched, a study identification number was created using a code for the site and a non-identifiable numeric code that was not linked to the original patient number. The link between the patient identification number and the study identification number was recorded in a study enrolment log. The log and the completed data collection forms were kept at the facility in a locked location with controlled access. Following study completion, these documents were destroyed. Data from the paper-based tool were entered into a password protected database using the generated study identification number, which were then imported into SAS for statistical analysis. Data were analysed at the EGPAF/Lesotho office in Maseru and EGPAF Global in Washington, DC.

Steps along the EID pathway were categorized into five stages. Stage 1 was the time from specimen collection to transfer to district laboratory. Stage 2 was the time from specimen receipt in the district hub to transfer to central laboratory for testing. Stage 3 was time from receipt of specimens at the central laboratory to receipt of results at district hospital. Stage 4 was the time from receipt of results at the district hospital to receipt at health facility. Stage 5 was the time from receipt of results at health facility to results receipt by caregiver.

For this study, we conducted an exploratory data analysis using numerical and graphical methods to display important features of the data, an analysis for each objective and its associated variables, and a descriptive data analysis. Exploratory data analysis allowed us to highlight general features of the data to direct future analyses and identify problem areas in the data.

The average–geometric mean (95% CI)–time intervals (in days) were stratified by relevant independent variables. They were calculated with linear mixed models assuming a compound symmetry working correlation structure to account for the clustering of women in facilities in order to determine the stages with the longest days.

In order to explore variations in TAT at study sites, for the purpose of this study, mean TATs (based on TAT for the caregiver) was categorized as long, (median 75.5–99 days), medium (62.5–67 days), or short (33.5–60 days).

Since this study was a retrospective review of existing records, there was no consent involved and the ethics committee waived the need for consent. This study received approval from the Baylor College of Medicine Children’s Foundation, Lesotho institutional review board and the Lesotho national research and ethics committee.

## Results

### Characteristics of the study population (mothers and infants)

In this study, 1187 HIV-exposed infants’ records were reviewed. The mean (standard deviation, SD) maternal age at the time of the delivery was 28 (±5.6) years.

Table 1 describes characteristics of the mothers and infants whose records were reviewed in this study. 92.8% of women received antiretroviral (ARV) drugs for PMTCT or treatment: 33.4% received antiretroviral therapy (ART) for their own health; 44.2% received zidovudine for prophylaxis against transmission; and 15.2% received an unknown ARV regimen. Maternal death rate at the time of review was 6.9% (82/1187).

**Table 1:** Mother and infant characteristics.

	Number of HIV exposed infants n (%) N = 1187
**Facility location (region)**	
Highlands	183 (15.4)
Foothills	528 (44.5)
Lowlands	476 (40.1)
**Maternal ART regimens during pregnancy**	
ARV for prophylaxis	525 (44.2)
ART prior to index pregnancy	161 (13.6)
ART initiated during index pregnancy	156 (13.1)
None	86 (7.2)
ART unknown when initiated	79 (6.7)
ARV regimen unknown	180 (15.2)
**Mothers alive**	
No	82 (6.9)
Yes	1023 (86.2)
Unknown	82 (6.9)
**Infant EID results at 6–8 weeks of life**	
Positive	47 (3.9)
Negative	1139 (95.9)
Unknown	1 (0.02)
**Infant received ARV prophylaxis after birth**	
Yes	1033 (87.0)
No	53 (4.5)
Unknown	101 (8.5)
**Infant alive**	
No	17 (1.4)
Yes	1089 (91.8)
Unknown	81 (6.8)
**HIV-infected infant initiated on ART**	N = 47
Yes	36 (76.6)
No	3 (6.4)
Unknown	8 (17.0)

Calculated interclass correlations (ICCs) for all variables and all were close to zero. These variables are not facility dependent. **Note:** ART: antiretroviral therapy; ARV: antiretroviral; EID: early infant diagnosis.

The mean (SD) infant age at blood draw was 46.9 (3.4) days. The HIV transmission rate at 6–8 weeks in the study participants was 3.9%. Of the 47 children who were HIV-infected, 39 had records of ART status, with 36/47 (76.6%) initiated on ART. The 36 children were initiated on ART at an average of 1.3 days (95%CI: 0.3–5.7; range: 10–56) after result receipt (in some cases children who tested positive were tracked outside the conventional system and contacted by phone, with some initiated on ART before they received the physical result through the conventional system). Overall 17/1187 (1.4%) of children had died at the time of review. The HIV status of children who died was unknown.

### Turnaround time analysis

S1 Fig shows mean times that specimens and results took at each stage of the EID cascade. Overall, the mean total turnaround time in this sample of sites was 61.7 days (95%CI: 55.3–68.7). The longest time spent by the specimen and results occurred at stage 3, which is time from receipt of specimen at the central laboratory to receipt of results at district hospital, and was 23.2 days (95%CI: 18.7–28.9). The average stage 1 and stage 3 time intervals were significantly shorter in the Lowlands Region (0.9 and 16.2 days), compared to Highlands Region (6 days [*P* = 0.03] and 34.3 days [*P* = 0.00].

The mean turnaround time was 47.4 (95%CI: 39.0–57.7) and 62.4 (95%CI: 55.6–69.9) for HIV-infected and HIV-uninfected children, respectively (Table 2). The mean (95% CI) turnaround time (in days) calculated from a linear mixed model including test result, region and facility level (Table 3), was found to be significantly shorter for HIV-infected infants compared to HIV-uninfected infants (p<0.01).

**Table 2:** Turnaround time (TAT) for the stages in the EID cascade calculated from linear mixed models.

Stage	Start time	Stop time	Infants HIV infected at 6–8 wks		Infants HIV uninfected at 6–8 wks		All infants at 6–8 wks	
			N (%)	Mean TAT in days (95%CI)	N (%)	Mean TAT in days (95%CI)	N	Mean TAT in days (95%CI)
1	Specimen collection	Specimen transfer to district laboratory	43 (4.0)	14.4 (11.3–18.4)	1045 (96.0)	13.9 (12.0–16.2)	1088	14.0 (12.1–16.1)
2	Specimen transfer from district laboratory	Specimen receipt at the central laboratory for testing	8 (3.2)	3.5 (0–15000)	239 (96.8)	2.0 (0.0–552.6)	247	2.0 (0.8–5.2)
3	Specimen transfer to central laboratory	Time results transferred from central laboratory to receipt of results at district hospital	16 (3.0)	20.6 (13.3–31.7)	510 (97.0)	23.2 (18.4–29.4)	526	23.3 (18.7–29.0)
4	Result receipt at district hospital	Receipt of results at health facility	19 (3.7)	3.0 (1.2–7.9)	494 (96.3)	3.2 (1.8–5.8)	513	3.2 (1.9–5.5)
5	Result receipt at health facility	Result receipt by caregiver	37 (4.2)	4.3 (2.4–7.5)	842 (95.8)	10.8 (8.2–14.2)	879	10.4 (7.9–13.5)
Overall	Specimen collection	Result receipt by caregiver	37 (4.2)	47.4 (39.0–57.7)	850 (95.8)	62.4 (55.6–69.9)	887	61.7 (55.3–68.7)

**Table 3:** Turnaround time for 887 subjects with complete data calculated from a linear mixed model including test result, region and facility level.

Variable	Number N = 887 n (%)	Turnaround time (days) Geometric mean	95%CI	p-value
**Test Result**				0.0036
**Positive**	37 (4.2)	47.1	(39.0–57.0)	
**Negative**	850 (95.8)	62.0	(55.9–68.8)	
**Region**				0.057
**Highlands**	77 (8.7)	63.6	(51.8–78.1)	
**Foothills**	401 (45.2)	54.6	(46.0–64.8)	
**Lowlands**	409 (46.1)	45.5	(37.3–55.5)	
**Facility Level**				0.21
**Hospital**	710 (80.0)	57.7	(48.9–68.2)	
**Health centre/filter clinic**	177 (20.0)	50.6	(43.1–59.4)	

Overall, the turnaround time of results based on geographical regions (Highlands, Foothills and Lowlands) or between hospitals and health centers varied but did not reach statistical significance.

## Discussion

In this study we found that the major contributor to health system delays in turnaround time in the EID cascade in Lesotho was the time from receipt of specimen at the central laboratory to return of results to district laboratory (Stage 3), followed by the time for transfer of specimens from health center to district laboratory hub (Stage 1). Although HIV-exposed infants are brought to the clinic at an average of seven weeks of life to have blood drawn for their 6-weeks DNA-PCR EID test, it took over two months for their caregivers to get the results back. In our step-by-step analysis, the longest delay occurred within the laboratory chain. In fact, even though it took two weeks to get the DBS samples from the collection point to the national central laboratory, it took more than three weeks to get the results back to district laboratories. This may be caused by the system focusing primarily on getting blood specimens from HIV-exposed infants while neglecting that the EID cascade is only complete when results get back to the caregiver. The main bottleneck in the EID cascade was the central laboratory.

Our data are similar to the findings of a study in Uganda, where a median turnaround time of EID at 39 days was found [13]. Together these findings point out that scale up of HIV services is not always accompanied by health system strengthening to absorb the additional burden that may occur with scale-up, including the laboratory system [14,15]. The effectiveness of PMTCT can only be achieved if HIV-exposed infants’ caregivers get results of their HIV status in a timely manner and children who are HIV-infected are initiated on ART [16–18]. In addition, children who are not HIV- infected at this early stage can continue to be monitored while they receive the much needed child survival package to reduce the risk of death from other childhood illnesses.

In our cohort of 6–8 weeks’ old infants, the HIV prevalence was below the targeted HIV MTCT-elimination rate of 5%. This result can be attributed to significant coverage of interventions for PMTCT in this study population, with over 90% coverage of maternal and neonatal ARV prophylaxis or treatment. This is encouraging but must be interpreted with caution. In a population where majority of the women breastfeed their infants such as in Lesotho, it is recognized that a significant number of children may still get infected throughout breastfeeding [15,19,20]. In April 2013, Lesotho adopted lifelong ART for all HIV-infected pregnant and breastfeeding women regardless of CD4 cell count or WHO clinical staging, ahead of the June 2013 WHO guidelines for PMTCT [9, 21]. The gains of this kind of policy change can only become a reality if the health system, including the laboratory network, is strengthened to ensure timely collection and processing of EID specimens and timely return of results to caregivers [20,22].

Our data demonstrated that, when the infants’ HIV status was considered, HIV-infected infants received their results in significantly shorter time. In addition, health care workers were requested to reach the caregivers of children who have a positive HIV DNA-PCR EID test result directly by phone. Children with positive results were required to be seen and initiated on treatment within seven days after communicating with the caregiver. Importantly, many of the children were initiated on treatment after the health facilities received their electronic results, well ahead of receiving the printed copies of the results. As part of overall program implementation, there was provision of mobile internet for electronic transfer of the positive DNA-PCR EID test results. There was no significant difference in turnaround time based on health facility level.

The limitations of this study included the fact that it was a retrospective cohort descriptive study. The use of existing medical records with incomplete information made data collection challenging.

Interventions to improve the delays have potential multiple benefits, which include improvement in the uptake of pediatric care and treatment for infants identified as infected, and ultimately contribute to reducing HIV morbidity and mortality in children. In addition, early knowledge of child’s status may serve a psychological boost for the mother to maintain maternal adherence to ART leading to better maternal health and adherence to required health care visits for HIV-uninfected children.

## Conclusion

In conclusion, the central laboratory and district laboratory were major contributors to the delays in TAT of initial EID in Lesotho. All geographical regions (highlands, foothills, and lowlands) and hospitals, and health centers were affected in a similar way. As technologies evolve with the advent of point-of-care testing (POCT) EID assays becoming available, it will be essential to evaluate how the new POCT approach impacts on the EID cascade. Furthermore, countries with similar challenges should consider efforts to improve timely initiation of ART for HIV-infected children and delivery of negative results to caregivers or providers.

## Supporting information

S1 Fig Mean turnaround time (TAT) (days) per stage. Stage 1: Time from specimen collection to transfer to the district laboratory: 14.0 days (95%CI: 12.1–16.1) Stage 2: Time from specimen transfer from the district laboratory to the central laboratory for testing: 2.0days (95%CI: 1.5–4.9) Stage 3: Time from receipt of specimens at the central laboratory to the time results transferred from central laboratory to receipt of results at district hospital: 23.2 days (95%CI: 18.7–28.9) Stage 4: Time from receipt of results at the district hospital to receipt at health facility: 3.3 days (95%CI: 1.9–5.5) Stage 5: Time from receipt of results at health facility to results receipt by caregiver: 10.4 days (95%CI: 7.9–13.5) Total time: 61.7 days (95%CI: 55.3, 68.7).
(TIF)

S1 File EID dataset.accdb. (ZIP)

S2 File Facility information.accdb. (ZIP)

S3 File Revised data collection tool for abstraction. (DOCX)

S4 File Revised HF Characteristics data collection tool. (DOCX)
