# Supplementary figures and images for: Conventional early infant diagnosis in Lesotho from specimen collection to results usage to manage patients: Where are the bottlenecks?
Source: PLoS One. 2017 Oct 10;12(10):e0184769. doi: 10.1371/journal.pone.0184769 (PMC5634554; doi:10.1371/journal.pone.0184769)

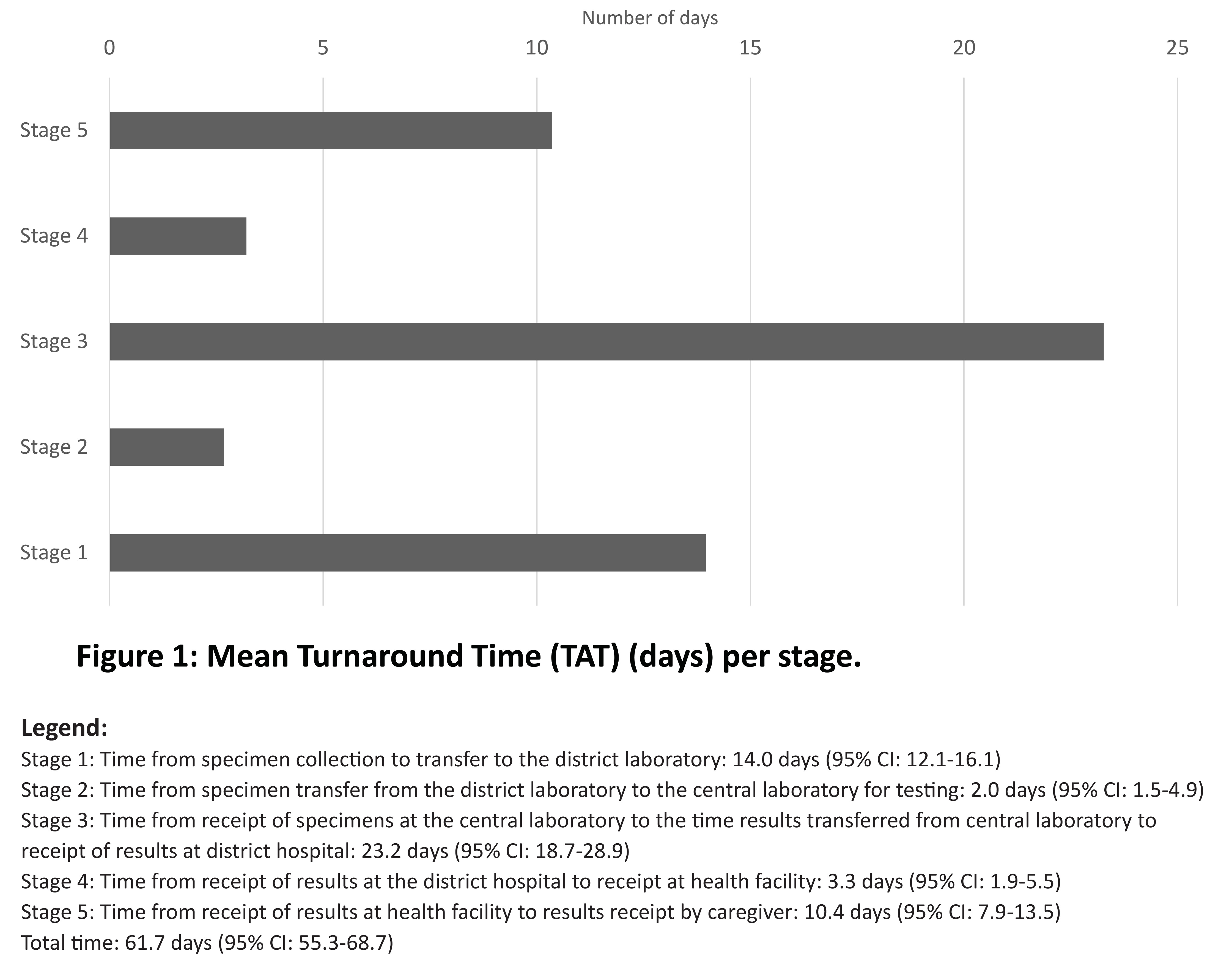

Supplement: S1 Fig — Stage 1: Time from specimen collection to transfer to the district laboratory: 14.0 days (95%CI: 12.1–16.1) Stage 2: Time from specimen transfer from the district laboratory to the central laboratory for testing: 2.0days (95%CI: 1.5–4.9) Stage 3: Time from receipt of specimens at the central laboratory to the time results transferred from central laboratory to receipt of results at district hospital: 23.2 days (95%CI: 18.7–28.9) Stage 4: Time from receipt of results at the district hospital to receipt at health facility: 3.3 days (95%CI: 1.9–5.5) Stage 5: Time from receipt of results at health facility to results receipt by caregiver: 10.4 days (95%CI: 7.9–13.5) Total time: 61.7 days (95%CI: 55.3, 68.7). (TIF) [file pone.0184769.s001.tif]
