# Supplementary material for: Conventional early infant diagnosis in Lesotho from specimen collection to results usage to manage patients: Where are the bottlenecks?
Source: PLoS One. 2017 Oct 10;12(10):e0184769. doi: 10.1371/journal.pone.0184769 (PMC5634554; doi:10.1371/journal.pone.0184769)
Supplement: S3 File — (DOCX) [file pone.0184769.s004.docx]

**Towards Getting More HIV-Positive Infants on Lifesaving Treatment: A Review of the Early Infant Diagnosis system, results, and outcomes in selected sites in Lesotho**

**Data Collection Tool for Abstraction**

*Date: 10 March 2012, Version: 4.0*

Completed by: ____________________________ Initials **__ __** Date completed: ___ /___ /____

dd/mm/yy

Facility: ______________________________ **__ __** **__ __ __ __** District: _________________________

Name Facility Code

***The study identification number is comprised of a two-letter code for the facility (different from the standard 5-alpha-numeric facility code) and a three-digit number. Look in the data collection SOP to locate the two-letter code for the study facility and start with 000 for the first record. Subsequent records should be (001, 002, 003…999). When starting at a new facility, begin again with 000 as the first record.***

Study identification number: **__ __ -** __ __ __

Reviewed by: ________________________________ Date completed: ___ /___ /____ dd/mm/yy

***Confirm eligibility criteria for record as found in EID database. If record does not meet both criteria, do not include the record. If the test date was in 2011 and no age was provided for the child, do not include in the study since eligibility cannot be determined, but keep a running tally on the CRF checklist in the space provided for this purpose.***

| Confirm eligibility criteria | |
| --- | --- |
| Age of child is one of the following (depending on how it appears in database): | 🞏 Age in days: 42-56d  🞏 Age in weeks: 6-8w |
| DBS-PCR test was done between 1^st^ of January 2011 – 31^st^ of December 2011 (date first specimen collected) | 🞏 Yes 🞏 No |

**Step 1: EID Database**

Completed by: ____________________________ Initials **__ __** Date completed: ___ /___ /____

dd/mm/yy

***Please complete all dates as day/month/year and tick ‘missing’ when there is no date available. The child’s name will be your primary means of linking data from this database to data in other registers/sources. Do not write the name down, but use the EID database or your printout of the records from the database to find the information needed to complete subsequent steps of this tool. Note that the patient identification number beginning with PCR (PatID) is listed in this database as well as printed out on a sticker with a bar code and found on the lab requisition forms maintained in MCH. If there are duplicate or misspelled names, this number may be useful to help verify that the appropriate child’s record has been located.***

| 1. Ward   *Provide the ward exactly as it appears in the database and select whether it is the ward name or code.* | Ward : **_______________________**  🞏 (1) Ward Code  🞏 (2) Ward Name |
| --- | --- |
| 1. Facility | Facility Code: **________**  Facility Name: **______________________** |
| 1. Hospital laboratory (hub) name | Hospital lab: _______________________ |
| 1. Age of child   *Select either days or weeks as it appears in the database. This should typically be in days.* | Age: __ __  🞏 (1) In days 🞏 (2) In weeks |
| 1. Date test received at headquarters | **__ __** /**__ __** /**__ __** |
| 1. Date sample was sent to SA NICD or Maseru Central Laboratory | **__ __** /**__ __** /**__ __** |
| 1. Date sample tested at SA NICD or Maseru Central Laboratory | **__ __** /**__ __** /**__ __** |
| 1. Test result   (Infant’s HIV status at 6-8 weeks of age) | 🞏 (1) Positive 🞏 (2) Negative  🞏 (3) Indeterminate 🞏 (4) No result generated |

**Step 2: DNA-PCR Register**

***Use child’s name from the EID database to link to his or her data in the DNA-PCR register. Since the registers are organized chronologically, the test date from the EID database will help you identify where to look for the child’s name****.* ***Noting the caregiver’s name, infant birth date and the patient chronic care file number may be helpful to link data in the other registers for subsequent steps.***

Completed by: ____________________________ Initials **__ __** Date completed: ___ /___ /____

dd/mm/yy

| 1. Date first specimen collected | __ __ /__ __ /__ __  dd mm yy  🞏 (99) Missing |
| --- | --- |
| 1. Date result received from district lab   *This should be the date the MCH clinic received the result.* | __ __ /__ __ /__ __  dd mm yy  🞏 (99) Missing |
| 1. Date result provided to mother or other caretaker   *Select option (1) if “initiated on ART” is written in register or if child was assigned a Patient Chronic Care File Number, indicating ART initiation.* | __ __ /__ __ /__ __  dd mm yy  🞏 (1) Date missing, but child was initiated on ART  🞏 (99) Missing |
| 1. Is there any indication provided in this register that the child has died? | 🞏 (1) Yes  If yes, provide the date if known:  __ __ /__ __ /__ __  dd mm yy  🞏 (0) No |
| 1. Under column on mother’s enrollment in ART services: is N/A selected, indicating that the mother has died? | 🞏 (1) Yes 🞏 (0) No 🞏 (99) Missing |

***(Step 2 continued) For infant PMTCT information, look first in the DNA-PCR register. If the information is not there, then check the EID database.***

| 1. Infant’s information was obtained from (source):   *Should use the DNA-PCR register to complete this step. Only use the EID database if the DNA-PCR record cannot be located. Do not use both sources, unless a reason is provided.* | 🞏 (1) DNA-PCR register  🞏 (2) EID database  🞏 (3) Information cannot be found in either source |
| --- | --- |
| 1. Did infant receive prophylaxis after birth? | 🞏 (1) Yes 🞏 (0) No  🞏 (9) N/A 🞏 (99) Missing |

**Step 3: Laboratory Reporting Form**

***Lab requisitions forms are typically found in MCH clinics in a folder or binder. The lab report from the laboratory that conducted the test should be stapled to the requisition form. The lab report form should either be from Maseru Central Laboratory or NICD in South Africa.***

Completed by: ____________________________ Initials **__ __** Date completed: ___ /___ /____

dd/mm/yy

| 1. Where was this sample tested? | 🞏 (1) Maseru Central Laboratory  🞏 (2) South Africa NICD  🞏 (3) Form not found  🞏 (99) Missing |
| --- | --- |

**Step 4: ANC Register (or the EID database or DNA-PCR register) - Mother PMTCT Information**

***Use child’s name and caregiver’s name (if mother) from DNA-PCR register to link to data in the ANC register (both registers kept at MCH). You may need to approximate when mother would have arrived in ANC based on test date (from EID database) to help you locate her record. Typically information for HIV-positive women is recorded in red, which will make this process easier.***

Completed by: ____________________________ Initials **__ __** Date completed: ___ /___ /____

dd/mm/yy

| 1. Mother’s information was obtained from (source):   *Should use the ANC register to complete this step. Only use the EID database or DNA PCR register if her ANC record cannot be located. Do not use more than one source, unless a reason is provided.* | 🞏 (1) ANC register  🞏 (2) EID database  🞏 (3) DNA-PCR register  🞏 (4) Information cannot be found in any source |
| --- | --- |
| 1. Mother’s ANC number   *Select N/A if using the EID database or DNA PCR register instead of the ANC register.* | __ __ __ __ __ __ 🞏 (9) N/A |
| 1. Mother’s age at first ANC visit (years)   *Select N/A if using the EID database or DNA PCR register instead of the ANC register. Select missing if using ANC register and age is not there.* | **__ __** 🞏 (9) N/A 🞏 (99) Missing |
| 1. Mother’s gravida   *Select N/A if using the EID database or DNA PCR register instead of the ANC register. Select missing if using ANC register and gravida is not there.* | **__ __** 🞏 (9) N/A 🞏 (99) Missing |
| 1. Mother’s parity   *Select N/A if using the EID database or DNA PCR register instead of the ANC register. Select missing if using ANC register and parity is not there.* | **__ __** 🞏 (9) N/A 🞏 (99) Missing |
| 1. Total number of ANC visits for this pregnancy   *Select N/A if using the EID database or DNA PCR register instead of the ANC register.* | **__ __** 🞏 (9) N/A |
| 1. Positive HIV status known before ANC visit   *Select N/A if using the EID database or DNA PCR register instead of the ANC register. Select missing if using ANC register and status before visit is not there.* | 🞏(1) Yes 🞏 (0) No  🞏 (9) N/A 🞏 (99) Missing |
| 1. ARV regimen | 🞏 (1) ARV prophylaxis  🞏 (2) ART prior to current pregnancy  🞏 (3) ART initiated during current pregnancy  🞏 (4) None  🞏 (99) Missing |
| 1. For women not on ART, was she eligible for ART?   *Select N/A if (1) the woman is on ART, (2) the woman’s status was negative in ANC, or (3) if using the EID database or DNA PCR register instead of the ANC register.*  *Select yes if woman was eligible, but not initiated.*  *Select no if woman was not eligible for ART.*  *Select unknown if woman’s HIV status is unknown and therefore wasn’t initiated on ART.* | 🞏 (1) Yes 🞏 (0) No 🞏 (3) Unknown  🞏 (9) N/A 🞏 (99) Missing |
| 1. For women initiated on ARV prophylaxis or ART during pregnancy: At what gestation was ARV prophylaxis initiated?   *Note that if a woman was initially started on prophylaxis, but was ART-eligible and started on ART during pregnancy, only the GA at which prophylaxis was given will be documented.* | **__ __** weeks gestational age  🞏 (9) N/A 🞏 (99) Missing |

**Step 5: ART Register – Infant ART Information**

Completed by: ____________________________ Initials **__ __** Date completed: ___ /___ /____

dd/mm/yy

***Use the child’s name to look up information in the ART register; the test date can be used to help locate the record in the register. The DNA-PCR register may contain the Patient Chronic Care Number (also known as the HIV Care/ART Card number), which can be used to locate the child’s ART card. ART cards may be kept in the MCH clinic (and not in the ART Corner) in some facilities.***

| 1. Do not complete this section if one of the following is true: | 🞏 (1) There are not ART services at this site  🞏 (2) The child is HIV-negative unless otherwise indicated in the DNA-PCR register (presumptive treatment)  🞏 (3) The child is deceased before initiation occurred |
| --- | --- |
| 1. Was the infant initiated on ART? | 🞏(1) Yes 🞏 (0) No |
| 1. Date of ART initiation:   *The date of ART initiation may also be found in the DNA-PCR register. Select N/A if child was not initiated on ART.* | **__ __** /**__ __** /**__ __**  dd mm yy  🞏 (9) N/A |
| 1. ART regimen: | 🞏 (1) AZT/3TC/Kaletra  🞏 (2) AZT/3TC/NVP  🞏 (8) Other, specify:________________ |

**Step 6: Hospital (district) Lab Improvised Register**

***Use child’s name from the EID database to link to the district lab improvised register. To help you identify where to look in the register for this information, use the date of blood draw/first specimen collected documented on this form in Step 2. Because these are improvised registers, the titles of the columns may vary from site to site.***

Completed by: ____________________________ Initials **__ __** Date completed: ___ /___ /____

dd/mm/yy

| 1. Date specimen is received at hospital laboratory (hub)   *This date may not be the same as the date first specimen was collected even within in the same hospital, but should be with a day. Specimens collected from the health center should be within a few days of this date.* | **__ __** /**__ __** /**__ __**  dd mm yy  🞏 (99) Missing  🞏 (9) N/A |
| --- | --- |
| 1. Date specimen sent to Maseru Central Laboratory | **__ __** /**__ __** /**__ __**  dd mm yy  🞏 (99) Missing |

| 1. Date result received at hospital laboratory (hub) | **__ __** /**__ __** /**__ __**  dd mm yy  🞏 (99) Missing |
| --- | --- |
