# Supplementary material for: Conventional early infant diagnosis in Lesotho from specimen collection to results usage to manage patients: Where are the bottlenecks?
Source: PLoS One. 2017 Oct 10;12(10):e0184769. doi: 10.1371/journal.pone.0184769 (PMC5634554; doi:10.1371/journal.pone.0184769)
Supplement: S4 File — (DOCX) [file pone.0184769.s005.docx]

**Towards Getting More HIV-Positive Infants on Lifesaving Treatment: A Review of the Early Infant Diagnosis system, results, and outcomes in selected sites in Lesotho**

**Health Facility Characteristics Data Collection Tool**

*Date: 10 March 2012, Version: 4.0*

Facility: ______________________________ Facility Code: **__ __ __ __ __**

District: ______________________________

Completed by: ________________________________ Initials **__ __** Date completed: ___ /___ /____

dd/mm/yy

1. Name of respondent: ________________________ Title: _____________________________ Date: ___ /___ /____

dd/mm/yy

1. Name of respondent: ________________________ Title: _____________________________ Date: ___ /___ /____

dd/mm/yy

1. Name of respondent: ________________________ Title: _____________________________ Date: ___ /___ /____

dd/mm/yy

Reviewed by: ______________________________________________ Date: ___ /___ /____

dd/mm/yy

| 1. **General facility information** | |
| --- | --- |
| 1. Facility level: | 🞏 (1) Hospital 🞏 (2) Filter clinic 🞏 (3) Health center |
| 1. Does this facility offer ART services? | 🞏 (1) Yes 🞏 (0) No |
| 1. Does this facility offer HIV pediatric treatment services? | 🞏 (1) Yes 🞏 (0) No |
| 1. Patient volume at this facility: | |
| - 1. How many new ANC clients attended this facility in 2011? | **__ __ __ __** |
| - 1. How many HIV-positive pregnant women attended this facility in 2011? | **__ __ __ __** |
| - 1. Of those HIV-positive women attending this facility in 2011, how many of them were transfers into the clinic during the women’s antenatal period? | **__ __ __ __** |

| 1. **HIV DNA-PCR sample and results processing and transport** | |
| --- | --- |
| 1. What is the hospital laboratory for HIV DNA-PCR blood samples (hub) for this facility? | 🞏 (1) Hospital laboratory name: _______________________________  Hospital code: __ **__** __ **__** __  🞏 (2) This facility is a hospital. |
| 1. How far away from the facility is the hospital laboratory (hub)?   *Only select “for the reviewers to complete” if you are at a hospital. Other facilities should complete both the time and distance away from the hospital laboratory.* | a. Time: __ **__** Hours, __ **__** Minutes  🞏 For the reviewers to complete  b. Distance: **__ __ __** Kilometers  🞏 For the reviewers to complete |
| 1. How far away from the hospital laboratory (hub) is Maseru Central Laboratory?   *Only select “for the reviewers to complete” if you are at a health center or filter clinic. Hospitals should complete both the time and distance away from the central laboratory.* | a. Time: __ **__** Hours, __ **__** Minutes  🞏 For the reviewers to complete  b. Distance: **__ __ __** Kilometers  🞏 For the reviewers to complete |
| 1. How are blood samples from HIV DNA-PCR infant testing transported to and from this facility to the hospital laboratory (hub) (check all that apply)?   *Only select “for the reviewers to complete” if you are at a hospital. Other facilities should tick all the modes of transport that apply.* | 🞏 (1) Vehicle 🞏 (2) Motorbike 🞏 (3) Bicycle  🞏 (4) Pony 🞏 (5) DHL pick-up  🞏 (8) Other, specify: _______________________  🞏 For the reviewers to complete |
| 1. How are blood samples from HIV DNA-PCR infant testing transported to and from the hospital laboratory (hub) to Maseru Central Laboratory (check all that apply)?   *Only select “for the reviewers to complete” if you are at a health center or filter clinic. Hospitals should tick all the modes of transport that apply.* | 🞏 (1) Vehicle 🞏 (2) Motorbike 🞏 (3) Bicycle  🞏 (4) Pony 🞏 (5) DHL pick-up  🞏 (8) Other, specify: _______________________  🞏 For the reviewers to complete |
| 1. How many days per week are blood samples from DNA-PCR infant testing transported from the facility to the hospital laboratory (hub)?   *Only select “for the reviewers to complete” if you are at a hospital. Other facilities should complete the number of days per week.* | Days per week: __  🞏 For the reviewers to complete |
| 1. How many days per week are blood samples from DNA-PCR infant testing transported from the hospital laboratory (hub) to Maseru Central Laboratory?   *Only select “for the reviewers to complete” if you are at a health center or filter clinic. Hospitals should complete the number of days per week.* | Days per week: __  🞏 For the reviewers to complete |
| 1. How many days per week are test results from DNA-PCR infant testing transported from Maseru Central Laboratory back to the hospital laboratory (hub)?   *Only select “for the reviewers to complete” if you are at a health center or filter clinic. Hospitals should complete the number of days per week.* | Days per week: __  🞏 For the reviewers to complete |
| 1. How often are test results from DNA-PCR infant testing transported from the hospital laboratory (hub) back to the facility? (select only one response)   *Only select “for the reviewers to complete” if you are at a hospital. Other facilities should complete the number of days per week.* | 🞏 (1) __ Day(s) per week  🞏 (2)__ Week(s) per month  🞏 (3) Once every month  🞏 (4) Once every 2 months  🞏 For the reviewers to complete |

| 1. **Identification/follow-up mechanisms** | |
| --- | --- |
| 1. Are there mechanisms in place at this facility for identifying HIV-positive mothers and HIV-exposed/infected infant pairs who fail to return to receive the infants’ EID results or whose results are returned early?   ***If no, skip Q15-Q19.*** | 🞏 (1) Yes 🞏 (0) No |
| 1. Are these mechanisms used to identify infants with an HIV-positive test result only or all exposed infants? | 🞏 (1) Infants with an HIV-positive result  🞏 (2) Infants with both results |
| 1. To what extent are **appointment books** used to identify HIV-positive mothers and HIV-exposed/infected infant pairs who fail to return to receive the infants’ EID results or whose results are returned early? | 🞏 (1) Always  🞏 (2) Most of the time  🞏 (3) Sometimes  🞏 (4) Rarely  🞏 (5) Never |
| 1. To what extent are **registers** used to identify HIV-positive mothers and HIV-exposed/infected infant pairs who fail to return to receive the infants’ EID results or whose results are returned early? | 🞏 (1) Always  🞏 (2) Most of the time  🞏 (3) Sometimes  🞏 (4) Rarely  🞏 (5) Never  If a response other than “never” was selected, specify which register(s): _________________________________  ________________________________________________ |
| 1. To what extent are **improvised books** used to identify HIV-positive mothers and HIV-exposed/infected infant pairs who fail to return to receive the infants’ EID results or whose results are returned early? | 🞏 (1) Always  🞏 (2) Most of the time  🞏 (3) Sometimes  🞏 (4) Rarely  🞏 (5) Never  If a response other than “never” was selected, specify which book(s):____________________________________  ________________________________________________ |
| 1. To what extent is **another method** used to identify HIV-positive mothers and HIV-exposed/infected infant pairs who fail to return to receive the infants’ EID results or whose results are returned early? | Specify method: __________________________________  🞏 (1) Always  🞏 (2) Most of the time  🞏 (3) Sometimes  🞏 (4) Rarely  🞏 (9) N/A |
| 1. Are there mechanisms in place at this facility for following up with defaulting mothers/caregivers who fail to return to receive the infants’ EID results or whose results are returned early?   ***If no, FINISH.*** | 🞏 (1) Yes 🞏 (0) No |
| 1. Are these mechanisms used to follow up infants with an HIV-positive test result only or all exposed infants? | 🞏 (1) Infants with an HIV-positive result  🞏 (2) Infants with both results |
| 1. To what extent are **SMS from the clinic** used to follow up with HIV-positive mothers and HIV-exposed/infected infant pairs who fail to return to receive the infants’ EID results or whose results are returned early? | 🞏 (1) Always  🞏 (2) Most of the time  🞏 (3) Sometimes  🞏 (4) Rarely  🞏 (5) Never |
| 1. To what extent are **phone calls from clinic** used to follow up with HIV-positive mothers and HIV-exposed/infected infant pairs who fail to return to receive the infants’ EID results or whose results are returned early? | 🞏 (1) Always  🞏 (2) Most of the time  🞏 (3) Sometimes  🞏 (4) Rarely  🞏 (5) Never |
| 1. To what extent are **village/community health workers** used to follow up with HIV-positive mothers and HIV-exposed/infected infant pairs who fail to return to receive the infants’ EID results or whose results are returned early? | 🞏 (1) Always  🞏 (2) Most of the time  🞏 (3) Sometimes  🞏 (4) Rarely  🞏 (5) Never |
| 1. To what extent are **support group members** used to follow up with HIV-positive mothers and HIV-exposed/infected infant pairs who fail to return to receive the infants’ EID results or whose results are returned early? | 🞏 (1) Always  🞏 (2) Most of the time  🞏 (3) Sometimes  🞏 (4) Rarely  🞏 (5) Never |
| 1. To what extent is **another method** used to follow up with HIV-positive mothers and HIV-exposed/infected infant pairs who fail to return to receive the infants’ EID results or whose results are returned early? | Method, specify: __________________________________  🞏 (1) Always  🞏 (2) Most of the time  🞏 (3) Sometimes  🞏 (4) Rarely  🞏 (9) N/A |
| 1. If village or community health workers (V/CHW) were selected above (Q23), please answer questions a-e. | |
| 1. Who is responsible for coordinating the follow-up conducted by the V/CHWs? | |
| 1. How many days are missed before follow-up with defaulting mothers/caregivers or when a result has been received early at the facility? | |
| 1. How many times do V/CHW attempt to locate the mother/caregiver (if they cannot find her the first time)? | |
| 1. How many V/CHW are attached to this facility? | |
| 1. Describe the challenges and benefits of this approach. | |
